# Content Validity and Reliability of the Pressure Ulcer Knowledge Test and the Knowledge Level of Portuguese Nurses at Long-Term Care Units: A Cross-Sectional Survey

**DOI:** 10.3390/jcm11030583

**Published:** 2022-01-24

**Authors:** Katia Furtado, Teresa Lopes, Anabela Afonso, Paulo Infante, Jaco Voorham, Manuel Lopes

**Affiliations:** 1 Out patient Department, Hospital of Portalegre, Unidade Local de Saúde do Norte Alentejano, 7300-312 Portalegre, Portugal; 2 Comprehensive Health Research Centre (CHRC), Universidade de Évora, 7000-671 Évora, Portugal; mlj@uevora.pt; 3 Emergency Department, University Hospitalar Center Cova da Beira, 6200-000 Covilhã, Portugal; aseret.lopes@gmail.com; 4 Department of Nursing, Health School, Polyctechnic Institute of Guarda, 6300-000 Guarda, Portugal; 5 CIMA, IIFA, Universidade de Évora, 7000-671 Évora, Portugal; aafonso@uevora.pt (A.A.); pinfante@uevora.pt (P.I.); 6 Departamento de Matemática, ECT, Universidade de Évora, 7000-671 Évora, Portugal; 7 DTIRS—Data to Insights Research Solutions, 1750-307 Lisboa, Portugal; jaco@dtirs.com; 8 São João de Deus School of Nursing, Universidade de Évora, 7000-671 Évora, Portugal

**Keywords:** knowledge, nursing, pressure ulcers, long-term care, Pressure Ulcer Knowledge Test, Portugal

## Abstract

(1) Background: Improvement in pressure ulcer care depends both on the dissemination of knowledge and its implementation. This study aims to translate the Pressure Ulcer Knowledge Test into Portuguese from Portugal and evaluate the internal consistency of the questionnaire. The second aim is to assess nurses’ pressure ulcer knowledge level. (2) Methods: The Pressure Ulcer Knowledge Test was translated into Portuguese, and the translated test’s internal consistency and content validity were assessed. Further, the authors conducted a cross-sectional survey using the test among 221 nurses working in long-term care units. (3) Results: The Cronbach’s alpha internal coefficient of reliability recorded for the 47 items was 0.738, which is higher than the minimum acceptable level of 0.7. The Cronbach’s alpha for the subscales was 0.709 for prevention/risk and less than 0.5 for staging and wound description. Only two of the 221 nurses achieved a score of 90% correct answers or more. The nurses scored lower in questions related to prevention/risk (Me = 67.4%, IQR = 60.6–75.8% vs. staging: ME = 85.7%, IQR = 71.4–85.7%, description: ME = 85.7%, IQR = 71.4–85.7%, *p* < 0.001). (4) Conclusion: The internal consistency of the instrument was acceptable. The instrument can accurately measure Portuguese nurses’ knowledge of pressure ulcers, and its information can help improve education and implementation of best practices.

## 1. Introduction

Pressure ulcers (PU) can be painful and negatively affect health-related quality of life and healthcare costs. Many people living in nursing homes and long-term care units are at risk of developing a PU due to age, immobility, and multiple comorbidities [1,2,3]. PU can lead to life-threating situation.

Despite increased attention over the past 20 years, formalised in international and national safety and quality health service standards [4,5,6,7], the prevalence of PU in Portugal has largely remained unchanged, while the associated costs of care continue to grow [8,9]. Reducing PU prevalence is a complex issue, requiring interventions that include education, evidence-based practice, enthusiastic implementation and auditing, resources, and multidisciplinary team involvement.

Nevertheless, it is widely accepted that when evidence-based interventions are implemented in clinical practice, it is possible to reduce the PU burden [10,11,12,13,14]. However, improvement in pressure ulcer care depends both on the dissemination of knowledge and its implementation.

In 2015, in the long-term care units (RNCCI) from Alentejo in Portugal, a working group was set up, and a trained facilitator was appointed to carry out a need assessment and design and implement a programme of improvement strategies to reduce healing time and prevent new wounds.

An initial prevalence study was carried out to assess the baseline dimension of the problem. Multiple interventions were designed based on the results obtained, including visits to each unit, wound assessments via telemedicine, education sessions, and bedside teaching. A point prevalence survey conducted three years later, using the same instrument in a homogenous group of patients, found that PU grades 3 and 4 continued to be the most prevalent complex wounds reported. PU represented 65.1% of all wounds [15] with poor healing rates. The literature shows that more severe stages are associated with a higher risk of complications such as critical colonisation, cellulitis, osteomyelitis, increased costs [16,17] and potential death.

Therefore, we decided to study eventual barriers to implementing best practice evidence among health care professionals working in long-term care units in Alentejo, starting by assessing knowledge of the prevention and treatment of pressure ulcers [18,19,20]. The Pressure Ulcer Knowledge Test (PUKT) [21] was chosen to assess PU knowledge among nurses working in long-term care settings. With objective information about nurses’ knowledge, we will be able to collect benchmark data, identify knowledge gaps, and implement educational strategies to improve clinical practice in Portugal’s long-term care settings.

People’s knowledge dramatically affects the safety, effectiveness, comfort, and satisfaction with which the goals of an individual or an organisation are formulated and attained knowledge influences people’s behaviour [22,23]. This indicates that nurses should have consistent knowledge of wounds and wound care to deliver evidence-based care. The literature has shown that nurses’ wound care knowledge, such as pressure ulcer knowledge, is limited and that education on wound care is unstructured at the undergraduate level and in continuing education [24,25].

It is necessary to assess nursing knowledge to know if the practice of nurses should be changed to minimise the prevalence of PU. With sufficient knowledge, both quality of care and patient safety can be improved [19,26,27,28,29,30,31,32]. Regular knowledge assessments are needed to gain insight into educational needs and priorities. Many instruments have been developed internationally to evaluate PU knowledge.

According to a recent systematic literature review [33], of the 18 instruments available, only five had been used more than once and were successful in a psychometric evaluation. The Pressure Ulcer Knowledge Test (PUKT) and the Pressure Ulcer Knowledge Assessment Tool (PUKAT) were the most valid and reliable instruments for measuring nurses’ wound care knowledge [33].

There is no valid and reliable instrument in Portugal to assess nurses´ knowledge of pressure ulcer prevention. This study aimed to examine the Portuguese version of the PUKT’s reliability by internal consistency and assess the level of knowledge of the nurses working in long-term care units. The PUKT was chosen because the instrument is relevant and acceptable to the study’s target group and is suitable for research purposes.

There is a lack of PU management research in long-term care settings. We hope that this study will help to emphasise that increasing knowledge is the basis for improving PU care and prevention to lead to quality and evidence-based practice.

## 2. Materials and Methods

### 2.1. Design and Ethical Considerations

A descriptive survey was conducted among nurses from all inpatient units of long-term care in Alentejo, Portugal, during May and June 2019. The survey was conducted online, and anonymity was guaranteed.

Formal authorisation to apply the instrument was obtained from the Board of Administration of Health in Alentejo (protocol code 27–02.03.2019). E-Informed consent was obtained from all subjects involved in the study.

### 2.2. Instrument

The Pieper–Zulkowski Pressure Ulcer Knowledge Test (PZ-PUKT) is one of the most used knowledge assessment tools [34,35,36,37,38]. The initial version, the Pressure Ulcer Knowledge Test (PUKT), developed by Pieper and Marvel in 1995, had three themes: prevention, classification, and wound description, with in total 47 items [21]. In 2014, Pieper and Zulkowski revised and updated the PUKT [35], and the number of items increased to 72. There are three subscales: prevention/risk (20 items), staging (25 items), and wound description (27 items), and it takes 20 to 30 min to complete. The reliability of the total PZ-PUKT (0.80) was strong, similar to that of the PUKT (0.85).

The PUKT was the instrument selected for this study because all the items are relevant and updated, and it takes less than 10 min to fill out. From our experience, this version will increase participant compliance, reduce dropouts and missing data compared to a more complex version, without compromising the power of the study.

Formal permission was obtained from the authors to translate the PUKT from English to Portuguese of Portugal, which two independent translators did. A 4-point Likert scale was used to evaluate the concordance rate between translators, one representing no concordance and four, total concordance. Total concordance was achieved for 42 items, and for five items, a score of 3 was achieved. Both translated versions were revised by three specialist nurses in wound care. A pre-test of the final instrument was applied to 10 nurses.

The PUKT Portuguese version, the instrument used in this study, consisted of two parts. Part one had ten items related to socio-demographic characteristics, one fewer than the original instrument, since race/ethnic background is not applicable in Portugal. Part two has 47 items that refer to prevention/risk (33 items), pressure ulcer staging (7 items), and wound description (7 items). Each item could be responded with true, false, or “I don´t know”. The correct answer to the questions is “true” in 34 cases and “false” in 13 (two about pressure ulcers, three about other ulcers, and eight about prevention/risk).

### 2.3. Data Collection

The instrument was sent to all nurses’ institutional emails by the coordinator of long-term care units in Alentejo, covering 550 nurses. Data entry of the PUKT results was done using SPSS 21.0 (IBM, Armonk, NY, USA).

### 2.4. Content Validity

To assess the content validity of the Portuguese version of the PUKT, 10 practice nurses with advanced experience with and knowledge of wound care, all members of the Portuguese Wound Care Association (ELCOS), evaluated the instrument. This evaluation focused on content validity in the Portuguese setting to identify gaps in the instrument, but also functioned as a pilot study to investigate the feasibility of our study design, participant recruitment, and collection of the study variables.

### 2.5. Statistical Analysis

The statistical analyses were done with the software R (version 4.0.3, R Core Team, Vienna, Austria). If a participant’s test had two or more missing answers in part 2, it was not included in the final analyses.

Reliability (internal consistency) by means of Cronbach’s alpha was measured for all items and by sub scale.

PUKT items were classified as incorrect whenever the participant responded “I don’t know” or did not respond. The number of correctly answered items of the PUKT was scored for the overall scale and each of the three subscales, and percentages were compared between categories of socio-demographic characteristics of the participating nurses.

Fisher’s exact test was used to assess the relationships in the classification of pairs of items, and as the measure of association between items, the Phi coefficient was calculated.

The Shapiro–Wilk test was used to assess the normality assumption of the overall and subscale scores. Levene’s test was used to assess the homoscedasticity of the scores. Due to the violation of the normality assumption, non-parametric tests were used. Friedman’s test was used to compare subscale scores, and when the null hypothesis was rejected, the Nemenyi multiple comparisons test was used. The Mann–Whitney test was used to compare scores between categories of dichotomous characteristics: gender, the time elapsed since reading the last article, and whether the nurses had already read the EPUAP/NPIAP/PPPIA guide. The Kruskal–Wallis test was used to compare scores between characteristics with more than two categories: professional category and time elapsed since attending training on pressure ulcers. When the null hypothesis of the Kruskal–Wallis test was rejected, the Nemenyi test was used for multiple comparisons. Significance was set at *p* < 0.05.

To assess the influence of possible confounders on the associations with the nurses’ performance, regression models were used that adjusted for the following nurse characteristics: sex, professional category, the time since reading a paper on pressure ulcers, the time since attending a lecture on pressure ulcers, and having read the EPUAP/NPIAP/PPPIA guidelines.

## 3. Results

### 3.1. Content Validity

The 10 specialised nurses who evaluated the validity of the Portuguese version of the instrument found it easy to fill out. Two nurses suggested minor changes in 3 and 4 questions, respectively, related to staging, which were accepted. It took all nurses less than 20 min to fill out the instrument, resulting in scores ranging from 78% to 95%.

### 3.2. Nurses’ Characterisation

Of the 550 contacted nurses, 221 (response rate of 40.2%) agreed to participate. More than 80% of the participants were female, 86% were general nurses, almost all (97%) were registered nurses, and none had a doctorate (Table 1). Participants were between 21 and 66 years old, with a mean age of 31.1 years (standard deviation [SD] 8.9). Although the number of years the participants have worked as a nurse ranged from 0 to 46, most had a short professional duration, the median (inter-quartile range) being 4 (2–9) years. The most common work environment was the Continuous Integrated Care Units (88.2%). Only eight participants indicated that they only worked in a single type of care, and 173 (78.3%) stated that they worked in two types of care. Three out of 5 (59.3%) participants attended a lecture on pressure ulcers during the previous year, and 84.2% read an article about pressure ulcers in the last year. More than half (55.2%) had not read the international guidelines set forth by the National Pressure Ulcer Advisory Panel/European Pressure Ulcer Advisory Panel (EPUAP/NPIAP/PPPIA).

### 3.3. Reliability

The Cronbach’s alpha internal coefficient of reliability, using the instruments filled out by the 221 participating nurses, for the 47 items was 0.738, which is higher than the minimum acceptable level of 0.7. Therefore, the internal consistency of the instrument was acceptable. The Cronbach’s alpha for the subscales were 0.709 for prevention/risk and less than 0.5 for staging and wound description.

### 3.4. Nurses’ Knowledge Level

**Figure 1:**
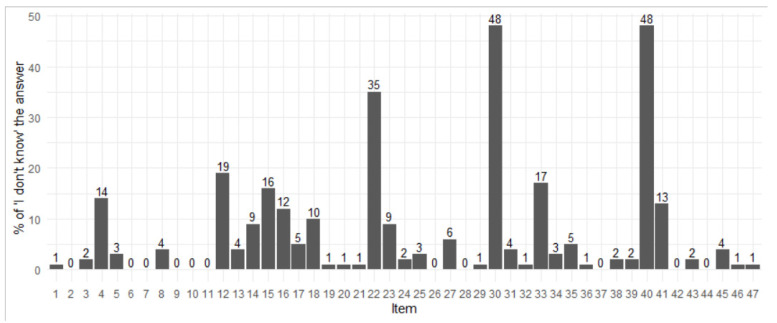
Percentage of participants who indicated not knowing the answer to the question. (Pressure ulcer stating scale items, Q1, Q6, Q9, Q20, Q32, Q37, and Q45; wounds description scale items, Q26, Q27, Q30, Q31, Q35, Q36, and Q44; and prevention/risk scale items, all others).

All the participants completed the PUKT-Portuguese version. The items with the highest percentages of “I don’t know” answers were in the prevention/risk subscale (Figure 1): “Vascular boots protect the heels from pressure” (Q40, 48.8%); “The incidence of pressure ulcers is so high that the government has appointed a panel to study risk, prevention, and treatment” (Q22, 34.8%); “A turning schedule should be written and placed at the bedside” (Q12, 19.0%); “A pressure relieving surface reduces tissue interface pressure below capillary closing pressure” (Q33, 16.7%); “In a side lying position, a person should be at a 30-degree angle with the bed” (Q15, 16.3%), and “Hot water and soap may dry the skin and increase the risk for pressure ulcers” (Q4, 14.5%). In the subscale wounds characterisation, 48.0% of nurses indicated that they did not know the answer to the item “Undermining is the destruction that occurs under the skin” (Q30).

The overall percentage of correct answers (score) ranged from 42.6 to 91.5, with a mean percentage of 71.2 (SD 8.2; Table 2). Only two respondents achieved a score higher than 90%. Of the 47 questions, 18 were answered correctly by 90% of the participants (pressure ulcers subscale, Q1, Q9, Q32 and Q37; Other Ulcers subscale, Q35, Q36 and Q44; prevention subscale, Q7, Q10, Q19, Q25, Q28, Q29, Q34, Q39, Q42, Q46 and Q47). The questions that less than 50% of the respondents answered correctly were: Q45 (48.4%) in the pressure ulcers subscale; Q30 (41.6%) in the other ulcers’ subscale; and Q18 (4.1%), Q23 (14.5%), Q17 (14.9%), Q40 (19.9%), Q11 (35.7%), Q13 (41.6%), Q4 (43.0%), Q5 (44.3%), and Q15 (44.3%) in the prevention/risk subscale.

The percentage of correct items differed significantly by subscale (*p* < 0.001, Table 2). Nurses had a significantly lower score in the prevention/risk subscale than in the other subscales (all *p* < 0.001).

### 3.5. Influence Factors of the Nurses’ Knowledge Level

A significant relationship was detected in PUKT scores between several pairs of items. In the pressure ulcers staging subscale, there is a moderate association between items 20 and 45 (phi = −0.20, Table S1). There is a strong association between items Q27 and Q31 (phi = 0.39) in the wound description subscale and a moderate association between items Q30 and Q35 (phi = −0.17, Table S2). In the prevention/risk subscale, there is a strong association between items Q13 and Q14 (phi = 0.49), and a moderate relationship between items Q5 and Q13 (phi = 0.32), Q7 and Q47 (phi = 0.31), Q39 and Q47 (phi = 0.31), and Q11 and Q17 (phi = 0.30, Table S3).

No significant differences were found between genders in PUKT scores, both overall and by subscale (all *p* > 0.05; Table 3). It was impossible to compare the scores by academic training because there was too little variation in the highest achieved education level; fewer than 9% of the participating nurses had more academic training than the 4-year nurse registration course. Significant differences were found in the prevention/risk scores by professional category; nurses with higher professional positions had higher scores than those with lower ones (Table 3). The percentage of correct answers, either globally or by subscale, did not differ significantly by the time elapsed either since attending a pressure ulcer training or since reading the last article on pressure ulcers. Nurses who had read the guide EPUAP/NPIAP/PPPIA had significantly higher PUKT total scores as well in the prevention/risk score subscale (Table 3).

The results from the adjusted multilinear and quasi-Poisson models were not relevantly different from the unadjusted ones and allowed to confirm the inexistence of measured confounding factors in the performed bivariate analyses.

## 4. Discussion

This study assessed Portuguese nurses´ knowledge of PU through a validated Portuguese version of the Pressure Ulcer Knowledge Test. The instrument was administered to 221 nurses working in long-term care settings. Our results show nurses’ lack of knowledge of pressure ulcers, especially regarding PU prevention.

All the obtained Cronbach’s alphas were lower than those presented by the original authors of this instrument [21,35]. The reliability of the total PUKT-PT is lower than what other studies reported with the same instrument: Cronbach’s alphas were between 0.83 and 0.93 for all items; 0.83 to 0.86 for the prevention, 0.82 regarding subscale staging, and 0.76 to 0.84 for wound description subscales [39,40,41]. As a further point of comparison, the validation study of PUKT in Brazil also obtained lower alphas regarding subscales for prevention, staging and wound description [38].

Our lower reliability scores in the sub-scores may be due to our limited sample size. Another factor that must be considered is that we have applied the test in a more heterogeneous population. In Portugal, nurses who work in long-term care are either inexperienced or work part-time combined with a second job. Furthermore, our study was multicentred, while previously published validations focused on hospital nurses [21,35,37,38,39,41].

Our findings pave the way to improve the internal consistency of this scale, an aspect already noticed by its original authors previously. They reported that as the test is used and the sample size increases, the reliability can be further examined, and test items refined [35].

A knowledge score of 80% was reported by the original authors of the PUKT instrument [35], higher than in our study, although other authors reported lower percentages, ranging from 65% to 69% [38,39,41]. An American investigation of nurses working in critical services noticed even lower results of 52%, although their sample had only 32 participants [36]. However, a study conducted in Iran found that the total PUKT score among critical nurses was higher than among nurses and students [37].

A meta-analysis reported that the nurses’ overall knowledge of PU prevention is lower than recommended levels, although it is higher than that of nursing students or assistant nurses [42]. A systematic review in different countries showed that nursing students do not have sufficient knowledge of PU prevention [31]. However, a Portuguese investigation about nursing students’ knowledge noticed that Portuguese nursing students showed adequate knowledge of aetiology, development, classification, risk assessment, nutrition, and PU preventive measures, influenced by the number of years in clinical practice and years of education [43].

Compared with the PUKT prevention results from other studies, which ranged from 69% to 77%, our prevention sub-scores were lower [35,38,39,41]. These results from our study may be influenced by the high frequency of junior nurses in our sample because 25% worked at most for two years and 50% at most for four years. However, previous investigations concerning the nurses’ overall knowledge level in preventing PU agree that it is extremely insufficient [44].

Regarding PU prevention, poor knowledge, inadequate training, physical skills, social influences, environmental context, high workload, shortage of resources (materials and staff, high rates of new, and substitute nurses), and inadequate communication were formerly identified as barriers [14,19,45].

Implementing pressure injury prevention programs reduced PU development and improved skills performance of nurses and multidisciplinary teams [14,46,47]. Training programmes centred on evidence-based nursing practices proved to increase tissue tolerance and significantly reduce tissue deterioration [48].

Regarding PU staging, the mean scores reported by the original authors of this instrument were higher, 86% [35], but other validation studies noticed lower results ranging from 67% to 70% [38,39,41]. Comparing our results from wound description subscales, we found a higher score than other studies that described mean scores between 59% and 77% [35,38,39,41].

Other studies recommended that improvement strategies should focus on enhancing nurses’ knowledge of the aetiology, development, classification, observation and risk assessment of PUs, nutrition plans, and preventive interventions [44]. Regular training courses and reviewing PU guidelines can be useful strategies to update nurses´ knowledge [42]. Nurses’ self-evaluations of their training needs can target education programs [30].

Implementing evidence-based practice programs also positively impacts staff satisfaction to achieve better outcomes, acquire quality improvement skills, competence, and confidence in wound management [46].

Despite these promising results, a previous systematic review noticed considerable uncertainty about whether educating healthcare professionals about prevention makes any difference to PU incidence or nurses’ knowledge of prevention because of the very low-certainty evidence provided by the included studies [32]. The authors noticed that only five randomised controlled trials were considered in the review, mainly with nurses [32]. Regarding the knowledge of pressure ulcers, it seems to us there is a lack of studies focusing on other healthcare professionals, namely physicians and physiotherapists. Even when looking at the available scales, reviews only reported tests measuring wound care knowledge of nurses [32,33].

Comparing median scores in the PUKT_PT with sociodemographic and educational data, only reading the EPUAP/NPIAP/PPPIA guideline was statistically significant for the global knowledge score. Another study also reported this finding [39], but not others [35,38,41].

Similar sociodemographic variables were found in validation studies of the PUKT instrument [35,38,39,41], excluding the Iranian validation that also included nursing students [37]. Educational variables from our study were different because, in Portugal, nurses have had a pre-graduation curriculum of four years since 1999. As a result, our graduation level is higher (91%) than in other studies [38,39].

Continuing professional development, reading articles or books, and attending lectures were more frequently reported than described in the literature [37,38,39,41]. Another study noticed that nurses who participated in PU training in the last two years had better PU prevention knowledge [30].

Only 45% of the participants read the EPUAP/NPIAP/PPPIA guideline. Other studies have also reported a low percentage, ranging between 19% and 50% [35,38,39,41]. We observed that participants who read the EPUAP/NPIAP/PPPIA guideline obtained significantly higher percentages of correct answers in the total score of PUKT and in the prevention subscale. A literature review about wound care evidence, knowledge, and education amongst nurses agrees with these findings: although empirical evidence is a standard of practice, nurses were also found to rely on scientific literature that is lower on the traditional hierarchy of evidence and additional types of information collected from informal sources [25]. Some reported barriers to implementing evidence-based practices in clinical settings were the poor application of knowledge, failure to apply recommendations from clinical guidelines, lack of awareness of protocols and clinical guidelines, and shortfalls in partnerships/integration between higher academic institutions and practice sectors [25].

This study has some limitations. First, the focus was on translation and adaptation of the questionnaire for use in Portugal. Therefore, the analysis of the translation’s psychometric properties was only preliminary. Second, the application of the PUKT to a sample of nurses working in long-term care was innovative. However, it hampers a comparison of our results with those from other studies. Another constraint that may have interfered with our results is that PUKT has a high number of questions, which has already been pointed out previously as possibly affecting the accuracy in answering the questions over time [37]. Finally, reported associations could have been caused by unmeasured confounders.

In conclusion, this study is the first to show that the PUKT is a useful instrument with good reliability to measure the knowledge of PU in Portuguese nurses working in long-term care settings. Results showed insufficiencies in the nurses’ knowledge of pressure ulcer prevention. Accordingly, in the future, the emphasis on education should be on prevention: prevention of initial tissue damage and prevention of progression of an ulcer to a more severe category. Nevertheless, educational interventions alone have little impact and should be embedded in a broader quality improvement bundle.

On the other hand, there is an increasing interest in using international benchmarking in terms of quality of services provided in long-term care settings. This tool, now validated for the Portuguese population, allows the recognition of the importance of knowledge of PU management, and should be applied nationally.

## Figures and Tables

**Table 1:** Socio-demographic characteristics of the participants (*n* = 221).

Variable	Category	N (%)
Gender	Female	181 (81.9)
	Male	40 (18.1)
Academic education	Registered nurse	202 (91.4)
	Master’s degree	7 (3.2)
	Other (bachelor, post-graduate, pro-graduate, specialty)	12 (5.4)
Professional category	Nurse	190 (86.0)
	Specialist nurse	13 (5.9)
	Responsible Nurse/Chief	18 (8.1)
Types of care in which you work	Continuous Integrated Care Units (UCCI)	195 (88.2)
	Medicine	19 (8.6)
	Surgery	16 (7.2)
	Urgency	13 (5.9)
	HC, OR/R, ICM, ICS, CIC, or Other	44 (19.9)
The last time attended a lecture on pressure ulcers	≤1 year	131 (59.3)
	2–3 years	59 (26.7)
	≥4 years	31 (14.0)
The last time read an article about pressure ulcers	≤1 year	186 (84.2)
	2–3 years	28 (12.7)
	≥4 years	7 (3.2)
Read EPUAP/NPIAP/PPPIA guidelines	Yes	99 (44.8)
	No	122 (55.2)

HC, Health centres; OR/R, Operating room/Recovery; ICM, Intensive care medicine; ICS, Intensive care surgery; CIC, Coronary intensive care; and EPUAP/NPIAP/PPPIA, National Pressure Ulcer Advisory Panel/European Pressure Ulcer Advisory Panel.

**Table 2:** PUKT scores by subscale.

Subscale	Range	Median (IQR)	Mean (SD)
Pressure ulcers staging	42.9–100.0	85.7 (71.4–85.7)	81.6 (12.7)
Wounds description	28.6–100.0	85.7 (71.4–85.7)	79.0 (15.2)
Prevention/Risk	33.3–87.8	66.7 (60.6–75.8)	67.4 (9.6)
47-item	42.6–91.5	72.3 (66.0–76.6)	71.2 (8.2)

IQR, Inter-quartile range; and SD, Standard deviation.

**Table 3:** Median (interquartile range) and significance test results, comparing the percentages of correct answers, globally and by subscale, between categories of socio-demographic characteristics of the participants.

Variable/Category	All Items		Subscale	
		Pressure Ulcers Staging	Wound Description	Prevention/Risk
**Gender**	*p* = 0.206 ^a^	*p* = 0.223 ^a^	*p* = 0.097 ^a^	*p* = 0.492 ^a^
Female	72.3 (68.1–76.6)	85.7 (71.4–85.7)	85.7 (71.4–85.7)	66.7 (63.6–75.8)
Male	70.2 (63.8–74.5)	78.6 (71.4–85.7)	78.6 (57.1–85.7)	66.7 (60.6–75.8)
**Professional category**	*p* = 0.081 ^b^	*p* = 0.795 ^b^	*p* = 0.692 ^b^	*p* = 0.038 ^b^
Nurse	72.3 (70.0–76.6)	85.7 (71.4–85.7)	85.7 (71.4–85.7)	66.7 (60.6–72.7)
Specialist nurse	74.5 (68.1–76.6)	71.4 (71.4–85.7)	85.7 (71.4–85.7)	66.7 (66.7–75.8)
Responsible Nurse/Chief	76.6 (70.2–80.3)	85.7 (71.4–85.7)	85.7 (71.4–85.7)	74.2 (66.7–78.8)
**When attended the last pressure ulcer training?**	*p* = 0.260 ^b^	*p* = 0.890 ^b^	*p* = 0.516 ^b^	*p* = 0.093 ^b^
≤1 year	72.3 (66.0–76.6)	85.7 (71.4–85.7)	85.7 (71.4–85.7)	66.7 (60.6–75.8)
2–3 years	70.2 (66.0–76.6)	85.7 (71.4–85.7)	85.7 (71.4–85.7)	66.7 (60.6–72.7)
≥4 years	76.6 (68.1–78.7)	85.7 (71.4–92.9)	85.7 (71.4–100.0)	72.7 (66.7–75.8)
**When read the last article on pressure ulcers?**	*p* = 0.423 ^a^	*p* = 0.762 ^a^	*p* = 0.342 ^a^	*p* = 0.764 ^a^
≤1 year	72.3 (68.1–76.6)	85.7 (71.4–85.7)	85.7 (71.4–85.7)	66.7 (60.6–75.8)
≥2 years	70.2 (66.0–75.5)	85.7 (71.4–85.7)	85.7 (57.1–85.7)	66.7 (62.1–72.7)
**Read** **EPUAP/NPIAP/PPPIA** **guidelines**	*p* = 0.001 ^a^	*p* = 0.231 ^a^	*p* = 0.077 ^a^	*p* = 0.001 ^a^
Yes	74.5 (69.1–78.7)	85.7 (71.4–92.9)	85.7 (71.4–85.7)	69.7 (63.6–75.8)
No	70.2 (63.8–76.6)	85.7 (71.4–85.7)	85.7 (71.4–85.7)	66.7 (60.6–72.7)

EPUAP/NPIAP/PPPIA-National Pressure Ulcer Advisory Panel/European Pressure Ulcer Advisory Panel. *p*-values obtained through ^a^ Mann–Whitney and ^b^ Kruskal–Wallis tests.

## Data Availability

Not applicable.

## Supplementary Materials

The following supporting information can be downloaded at: https://www.mdpi.com/article/10.3390/jcm11030583/s1, Table S1: Phi coefficient between items of ulcers staging subscale; Table S2: Phi coefficient between items of wound description subscale; Table S3: Phi coefficient between items prevention/risk subscale.
