# Supplementary material for: Content Validity and Reliability of the Pressure Ulcer Knowledge Test and the Knowledge Level of Portuguese Nurses at Long-Term Care Units: A Cross-Sectional Survey
Source: J Clin Med. 2022 Jan 24;11(3):583. doi: 10.3390/jcm11030583 (PMC8836429; doi:10.3390/jcm11030583)
Supplement: Supplementary file 1 [file jcm-11-00583-s001.zip › jcm-1506862-supplementary.pdf]

Supplementary Materials

Table S1. Phi coefficient between items of ulcers staging subscale.

Item	6	9	20	32	37	45
**1**	0.06	-0.03	0.11	0.02	-0.03	0.07
**6**		-0.08	0.01	0.05	0.09	-0.02
**9**			0.09	-0.03	-0.01	-0.04
**20**				0.11	0.01	-0.20
**32**					-0.03	-0.05
**37**						-0.12

Table S2. Phi coefficient between items of wound description subscale.

item	27	30	31	35	36	44
**26**	0.13	0.14	0.03	-0.07	-0.10	0.14
**27**		-0.06	0.39	0.04	-0.01	-0.06
**30**			0.02	-0.17	0.11	-0.08
**31**				0.13	0.05	-0.03
**35**					0.11	-0.02
**36**						-0.01

Table S3. Phi coefficient between items prevention/risk subscale.

Item	3	4	5	7	8	10	11	12	13	14	15	16	17	18	19	21	22	23	24	25	28	29	33	34	38	39	40	41	42	43	46	47
**2**	0.03	0.15	0.04	0.05	-0.01	0.18	0.07	0.11	-0.04	-0.03	0.07	0.07	-0.02	-0.15	0.14	0.27	0.06	0.01	0.10	0.14	0.09	0.05	0.05	0.24	-0.10	-0.05	0.06	0.07	-0.10	-0.05	0.05	-0.03
**3**		0.00	-0.10	-0.01	-0.01	0.02	-0.22	0.06	-0.06	-0.05	0.07	0.03	-0.20	0.00	0.12	0.00	0.13	-0.05	0.14	-0.02	-0.02	0.07	0.02	0.12	0.07	-0.07	0.03	0.02	0.01	-0.06	0.01	-0.08
**4**			0.18	0.07	-0.04	0.12	0.06	0.10	0.03	0.13	0.14	0.20	0.07	-0.04	0.01	0.06	0.13	0.24	-0.02	0.15	0.10	0.13	0.02	0.01	0.00	-0.05	-0.07	0.17	0.06	0.14	-0.01	0.08
**5**				0.07	-0.14	0.05	0.19	0.11	0.32	0.28	0.08	0.17	0.14	-0.05	0.02	0.07	-0.06	0.05	-0.09	0.02	0.03	0.13	0.10	0.02	-0.01	0.01	-0.13	-0.04	-0.10	0.04	0.00	0.09
**7**					-0.08	0.21	-0.01	-0.03	0.13	0.16	-0.05	-0.02	-0.02	-0.12	-0.03	0.13	0.06	-0.11	0.10	-0.03	-0.02	-0.04	0.00	-0.04	0.02	0.18	0.08	0.09	-0.05	0.09	0.27	0.31
**8**						-0.06	0.07	0.09	-0.23	-0.08	0.04	0.07	0.07	0.00	0.02	-0.15	0.06	-0.01	0.03	0.02	0.05	-0.02	0.00	0.04	-0.10	0.17	0.04	0.03	0.06	-0.07	0.00	0.01
**10**							0.03	0.07	0.05	0.00	0.05	0.08	0.06	-0.14	0.13	0.15	0.03	0.06	0.13	0.13	-0.02	-0.03	-0.07	-0.04	-0.06	-0.02	-0.02	0.05	-0.04	-0.02	0.19	-0.01
**11**								0.11	0.06	0.04	0.00	0.05	0.30	0.09	0.16	0.00	0.01	-0.15	-0.04	-0.02	0.01	0.01	0.06	-0.03	0.13	0.05	-0.09	0.05	0.05	0.12	-0.03	0.07
**12**									0.02	-0.02	0.05	0.09	-0.10	-0.07	0.18	0.09	0.24	-0.01	0.07	0.00	0.04	0.21	0.17	-0.01	0.00	0.09	0.17	0.13	0.07	-0.02	0.04	0.10
**13**										0.49	-0.05	0.08	0.06	-0.03	-0.04	0.07	0.01	-0.03	-0.14	0.05	0.02	0.16	0.08	0.00	-0.01	0.01	-0.15	0.05	0.05	0.02	0.01	-0.02
**14**											0.10	0.17	0.05	0.02	-0.04	0.04	0.06	-0.09	-0.07	0.01	0.04	0.13	0.15	0.03	0.08	0.04	-0.11	0.08	0.01	0.06	0.07	0.10
**15**												0.26	-0.09	-0.05	0.11	0.04	0.19	0.05	-0.01	0.06	0.03	0.05	0.15	0.09	-0.01	0.14	0.06	0.29	0.10	0.12	0.06	-0.01
**16**													0.10	0.07	0.17	0.06	0.07	0.07	0.00	0.17	-0.07	0.04	0.25	0.10	-0.07	0.19	0.09	0.06	0.05	0.08	0.03	0.16
**17**														0.11	0.03	0.01	-0.15	0.12	-0.17	0.09	0.05	-0.12	0.15	-0.16	0.00	0.06	-0.05	0.00	0.08	0.02	-0.04	0.04
**18**															0.04	-0.13	0.03	-0.08	0.03	-0.07	0.02	-0.05	0.05	0.06	0.09	-0.12	-0.05	-0.10	0.06	-0.10	-0.17	0.02
**19**																0.18	0.13	-0.03	0.14	0.06	-0.03	0.04	0.16	0.18	-0.03	0.11	0.05	0.03	-0.06	0.03	0.08	-0.02
**21**																	0.11	0.00	0.02	-0.02	-0.04	0.03	-0.02	0.04	0.03	0.03	-0.01	0.10	-0.01	0.03	0.12	-0.04
**22**																		0.10	0.08	0.13	-0.02	0.09	0.17	0.15	0.07	0.19	0.13	0.25	0.03	-0.01	0.03	0.12
**23**																			-0.07	0.03	-0.06	0.10	0.08	0.07	-0.15	-0.02	0.15	0.02	-0.07	-0.04	-0.05	-0.10
**24**																				-0.03	-0.05	0.01	0.06	0.06	0.03	0.10	0.06	0.14	-0.08	0.02	0.25	0.09
**25**																					-0.03	-0.05	0.22	0.02	-0.03	-0.03	-0.06	0.17	0.09	-0.01	0.01	-0.02
**28**																						-0.03	-0.06	-0.03	0.06	-0.02	-0.04	0.17	0.11	0.09	-0.04	-0.01
**29**																							0.18	0.08	0.01	0.10	0.07	0.08	-0.07	-0.08	0.13	-0.02
**33**																								0.07	0.01	0.15	0.11	0.13	0.02	0.04	0.08	0.19
**34**																									0.02	-0.04	0.02	0.05	-0.09	0.05	0.09	-0.03
**38**																										-0.06	0.02	0.13	-0.03	0.17	0.04	-0.04
**39**																											0.08	0.15	-0.05	-0.04	0.06	0.31
**40**																												0.02	-0.06	-0.06	0.00	0.05
**41**																													0.07	0.06	-0.06	0.03
**42**																														0.00	-0.09	-0.03
**43**																															0.02	0.04
**46**																																0.14
